# Bimodal Data Fusion of Simultaneous Measurements of EEG and fNIRS during Lower Limb Movements

**DOI:** 10.3390/brainsci11060713

**Published:** 2021-05-27

**Authors:** Maged S. AL-Quraishi, Irraivan Elamvazuthi, Tong Boon Tang, Muhammad Al-Qurishi, Syed Hasan Adil, Mansoor Ebrahim

**Affiliations:** 1 Department of Electrical and Electronic Engineering, Universiti Teknologi PETRONAS, Bandar Seri Iskandar 32610, Malaysia; eng.mgd@gmail.com (M.S.A.-Q.); irraivan_elamvazuthi@utp.edu.my (I.E.); 2 Faculty of Engineering, Thamar University, Dhamar 87246, Yemen; 3 Centre for Intelligent Signal and Imaging Research, Universiti Teknologi PETRONAS, Bandar Seri Iskandar 32610, Malaysia; 4 Faculty of information and Computer Science, Thamar University, Dhamar 87246, Yemen; mssqpr@gmail.com; 5 Faculty of Engineering, Sciences and Technology, Iqra University, Karachi 75500, Pakistan; hasan.adil@iqra.edu.pk (S.H.A.); mebrahim@iqra.edu.pk (M.E.)

**Keywords:** neurovascular coupling, fNIRS, EEG, lower limb movements

## Abstract

Electroencephalography (EEG) and functional near-infrared spectroscopy (fNIRS) have temporal and spatial characteristics that may complement each other and, therefore, pose an intriguing approach for brain-computer interaction (BCI). In this work, the relationship between the hemodynamic response and brain oscillation activity was investigated using the concurrent recording of fNIRS and EEG during ankle joint movements. Twenty subjects participated in this experiment. The EEG was recorded using 20 electrodes and hemodynamic responses were recorded using 32 optodes positioned over the motor cortex areas. The event-related desynchronization (ERD) feature was extracted from the EEG signal in the alpha band (8–11) Hz, and the concentration change of the oxy-hemoglobin (oxyHb) was evaluated from the hemodynamics response. During the motor execution of the ankle joint movements, a decrease in the alpha (8–11) Hz amplitude (desynchronization) was found to be correlated with an increase of the oxyHb (r = −0.64061, *p* < 0.00001) observed on the Cz electrode and the average of the fNIRS channels (ch28, ch25, ch32, ch35) close to the foot area representation. Then, the correlated channels in both modalities were used for ankle joint movement classification. The result demonstrates that the integrated modality based on the correlated channels provides a substantial enhancement in ankle joint classification accuracy of 93.01 ± 5.60% (*p* < 0.01) compared with single modality. These results highlight the potential of the bimodal fNIR–EEG approach for the development of future BCI for lower limb rehabilitation.

## 1. Introduction

Multimodal neuroimaging techniques have a major impact on neuroscience today. Such approaches have contributed independently to the comprehension of cognitive processing [1,2], and to the enhancement of clinical diagnosis [3,4]. Non-invasive neuroimaging techniques can be categorized according to their recording principles: electrophysiological measurement, such as magnetoencephalography (MEG) and electroencephalography (EEG); and hemodynamic measurement, including positron emission tomography (PET), functional magnetic resonance imaging (fMRI), and functional near-infrared spectroscopy (fNIRS). Individually, these modalities suffer from specific resolution drawbacks, e.g., the electrophysiological approaches suffer from spatial resolution, while the hemodynamic techniques suffer from the slow blood dynamic response that limits their temporal resolution [5]. The integration of multiple imaging modalities has been suggested to help overcome the drawbacks by complementing each other [6,7]. As a result, recent research has focused on combining electrophysiological and hemodynamic signals (fNIRS, BOLD) [8,9,10,11].

While the idea of integrating multiple modalities is not new, there remains a range of technical challenges. For instance, during concurrent fMRI–EEG recording, EEG data collected during fMRI acquisition could be affected by artifacts induced by gradients from magnetic fields. EEG–fNIRS-based integration is the most popular technique owing to its portability, low cost, and system’s flexibility [12]. The fNIRS is a non-invasive optical imaging technique that typically uses two or more different wavelengths to measure oxygenated hemoglobin (oxyHb) and deoxygenated hemoglobin (deoxyHb) concentration changes (between 600 and 1000 nm). These measures were similar to the response of fMRI to blood oxygen dependency (BOLD). The BOLD signal measured with fMRI is mainly prone to venous blood flow, whereas the fNIRS often detect hemodynamic changes at the capillary stage [13]. However, many aspects that potentially make fNIRS advantageous for coupling assessment with EEG than fMRI and positron emission tomography (PET) include size, portability, and low cost, as well as evidence that brain capillary oxygenation, which is observed by fNIRS, reflects neuronal activity to a large degree [13,14,15].

The integration processes can be categorized into three scenarios: predictive integration; constraint integration; and integration by fusion [13]. One of the significant multimodal approaches to neuroimaging is integration through prediction. An example of this scheme used temporally-resolved EEG signal as a predictor of changes in hemodynamic response recorded simultaneously [16,17]. The resulting region-specific hemodynamic correlation can then be identified using another neuroimaging modality that has high spatial resolution [18,19]. Several such studies such as [20,21] have focused on the correlation of modulations in ongoing EEG-measured oscillatory activity with the hemodynamic signal. In particular, these studies have shown that alpha rhythm modulations (oscillations at ~10 Hz) are negatively correlated with BOLD signal modulations, during motor execution task [22]. Simultaneous EEG and fNIRS measurements have been carried out by Takuro et al. [14] to demonstrate the relationship of the hemodynamic response and neural activities during motor preparation of hand movement. The results revealed a correlation between the readiness potential (RP) and the hemodynamic response in the premotor cortex contralateral to the performing hand. Another concurrent EEG and NIRS study was performed to investigate the relationship between brain rhythmic activity and the hemodynamic response during self-paced right finger movements. Person’s correlation was performed between the amplitude of event-desynchronization in the alpha band and the oxyHb in the motor cortex area [23]. Separately, integration of EEG with fNIRS by fusion has been proposed to improve the brain-computer interface (BCI) performance [5]. In summary, the previous studies utilizing the integration of EEG and fNIRS modalities focus on upper limb movements such as finger tapping, pushing a button, and left/right-hand movements; studies regarding lower limb movements are still rarely investigated.

According to the hypothesis stating that modulation in the alpha rhythm (~10 Hz) is negatively correlated with the hemodynamic response signal modulations, during motor execution task, this study aims to investigate the relationship between the electrophysiological activities and the hemodynamic response in the motor cortex area during a lower limb movement’s task—the ankle dorsiflexion movement. To the best knowledge of the authors, this is the first study that investigates the correlation between the alpha band with the hemodynamic response (oxy and deoxyhemoglobin) during ankle movements and exploits the fusion of the bimodality to detect ankle movements. Ankle joint movements were selected thanks to their important role in individual walking positions and posture [24].

## 2. Materials and Methods

**Figure 1:**
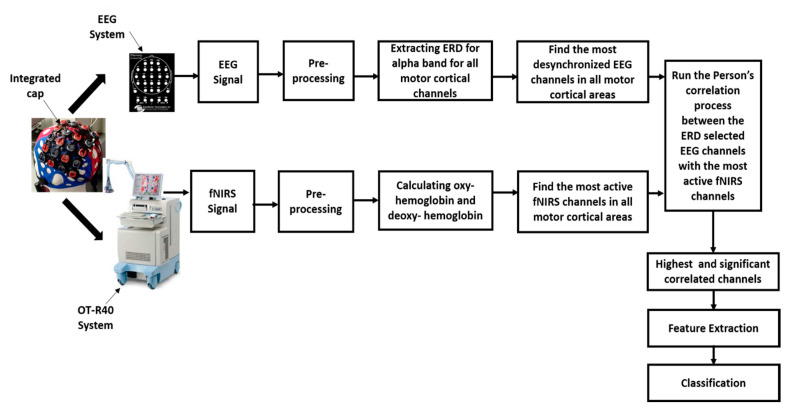
The proposed framework of functional near-infrared spectroscopy (fNIRS)–electroencephalography (EEG) simultaneous measurements and analysis during lower limb movements. ERD, event-related desynchronization.

The data collection and preprocessing procedures are outlined in this section. First, details about the participants are given, followed by the description of the implementation of different neuroimaging modalities. Then, the experimental protocol and method of data preprocessing are presented. Figure 1 illustrates the general framework of this experiment.

### 2.1. Participants

**Figure 2:**
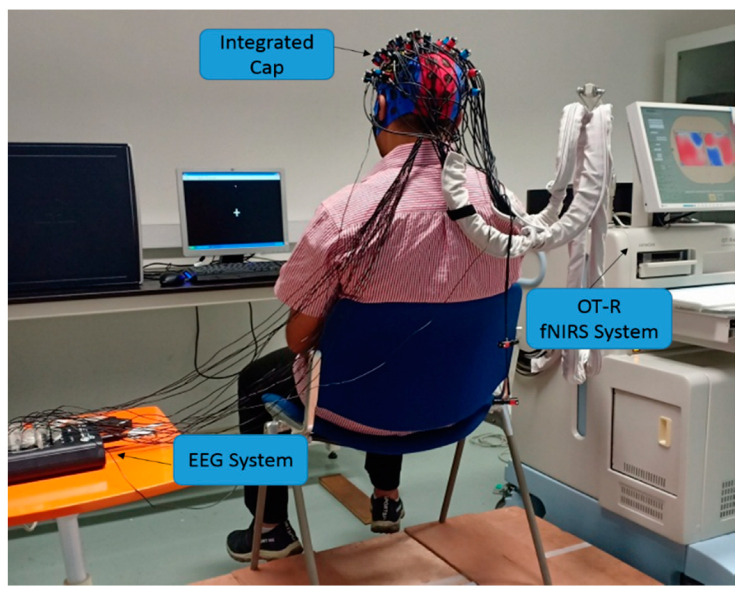
Subject position during the data collection process. EEG electrodes and fNIRS optodes connected to the integrated cap on the subject’s head, with the subject sat on a chair in front of an instruction monitor.

A total of twenty right-handed healthy male subjects (mean age 30.16 ± 5.35) participated in this experiment. They were recruited from the Universiti PETRONAS Teknologi (UTP) campus of undergraduate and postgraduate students. This research was approved by the Ethics committee of the Universiti Pendidikan Sultan Idris. The informed consent was given in writing by all volunteers. Before the data acquisition procedure, all the steps in the experiment were explained to the participants. The subjects were seated on a comfortable chair with their leg not touching the floor to ensure the full range of movement of the ankle joint and the knee joint was kept at 90°. The monitor was positioned around 85 cm in front of the subject to give the visual instruction of the task, as depicted in Figure 2. The entire experiment was carried out in accordance with the Declaration of Helsinki.

### 2.2. Procedure

**Figure 3:**
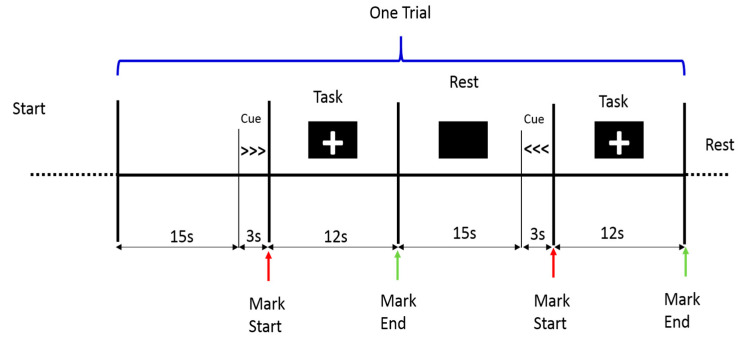
Experimental protocol timeline during the ankle joint movement. The trial started with 15 s rest until the cue signal appeared on the screen to indicate the right or left movements, then the subject moved his ankle for 12 s until the (+) sign disappeared.

The data acquisition experiment was carried out in a quiet room to minimize environmental disturbances. At least 5 min before the experiment, the participant was asked to stay relaxed before the task started. The trial started with a blank screen for 15 s, then a cue signal appeared for 3 s to indicate the direction of the movement (right or left ankle); next, the participants were allowed to move the ankle joint when a fixation cross appeared at the center of the black screen. The participants continued to move their ankles for 12 s until the fixation cross disappeared. Then, the participants were given 15 s to relax (rest period). The experimental protocol consisted of 10 trials of ankle dorsiflexion movement (12 s per movement task) with inter-trial intervals of 15 s. In addition, 3 s was set for movement cues to indicate the limb side that the participant should move (right or left ankle), as depicted in Figure 3. The data were collected in a single recording from each subject.

**Figure 4:**
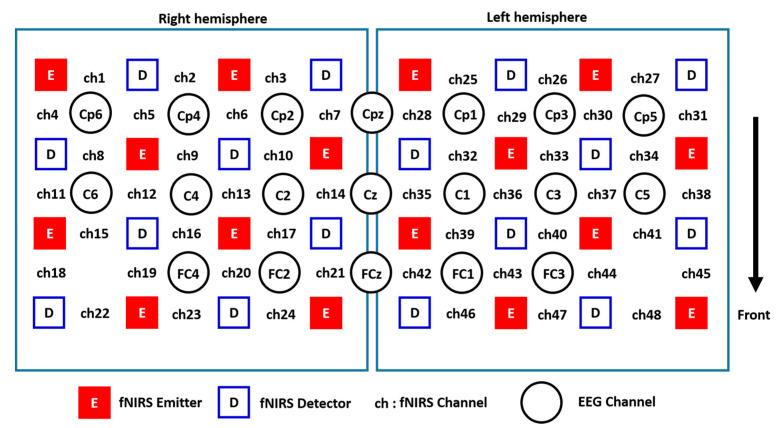
Distribution of the EEG electrodes and fNIRS optodes placed on both hemispheres of the motor cortex area.

Simultaneous fNIRS and EEG measurements were conducted during the experiment, by having the fNIRS optodes and EEG electrodes installed into a customized integrated EEG–fNIRS cap, MCScap (Medical Computer System Ltd., Moscow, Russia), as shown in Figure 2. In this experiment, we used 4 × 4 fNIRS probes for each hemisphere. The EEG electrodes were placed based on the 10–10 system, and the fNIRS optodes were positioned according to the Cz with an inter-optode distance of 3 cm between the emitter and detector. Hence, we had 48 fNIRS channels and 19 EEG channels (FCz, FC1, FC2, FC3, FC4, Cz, C1, C2, C3, C4, C5, C6, CPz, CP1, CP2, CP3, CP4, CP5, CP6) distributed over the motor cortex areas, as depicted in Figure 4. An fNIRS channel (Ch) is defined as the measuring area between a pair of emitter-detector optodes. The control of simultaneous measurements was managed using MATLAB custom code (MathWorks, Natick, MA, USA), which sent triggers to the EEG system and the fNIRS system via parallel and serial ports to mark the start and end of the task in each trial of the movement task.

### 2.3. EEG Data Acquisition Procedure

The EEG signals were recorded during the ankle joint movements using BrainMaster Discovery 24E (BrainMaster Technologies Inc., Bedford, OH, USA) at a sampling frequency of 256 Hz [25], with the reference left and right ear lobe (A1–A2) and the ground anterior to Fz positioned according to a 10/10 system. The impedance of the electrodes was maintained below 5 k ohm.

### 2.4. fNIRS Data Acquisition Procedure

Concentration changes of oxyhemoglobin (oxyHb) and deoxyhemoglobin (deoxyHb) in bilateral motor cortices, which reflect the hemodynamic response, were recorded using a multichannel NIRS unit operating at wavelengths of 695 nm and 830 nm (OT-R40, Hitachi, Japan), with a sampling frequency of 10 Hz [25].

### 2.5. EEG Data Processing

The EEG signals were first filtered using a bandpass filter (0.05–40 Hz) using a finite impulse response (FIR) filter. Then, the processed data were segmented into 22 s segments: 4 s prior and 18 s post movement’s onset. Independent components analysis (ICA) was carried out on the segmented data to eliminate visible artifacts such as ocular movements, cardiac signal, and muscle contraction. ICA components with these artifacts were removed, and the remaining components were projected back to rebuild the EEG signals. Time-frequency representation of the EEG channels was evaluated before extraction of the ERD feature. Thus, wavelet transform analysis was implemented in this work using complex Morlet wavelet convolution. The parameters of the complex Morlet wavelet convolution were set with minimum and maximum frequencies of 5 Hz and 40 Hz, respectively. The number of frequencies was set as 20 and the wavelet cycle was chosen as five cycles [26]. Thereafter, processed EEG signals were passed through the wavelet transformation to obtain the time-frequency representation (TF). In order to extract power spectral density (PSD) in the alpha (8–11 Hz) band, a fast Fourier transform (FFT) was implemented to non-overlapping segments and then averaged across segments under the same conditions. A Hamming window was employed to control the spectral leakage [27]. An event-related (de)synchronization (ERD/ERS) technique was utilized to quantify the event-related in relation to EEG power changes. The ERD/ERS transition was defined as the percentage of power density decrease/increase over the task with respect to the baseline value (rest condition) [28]. ERD was evaluated according to Equation (1):

(1) $$ERD=\frac{P-R}{R}\times 100$$

where *P* is the power within the alpha frequency band in the period during the movement task, whereas *R* is the reference or the baseline prior to the movement.

### 2.6. fNIRS Data Processing

The optical signals from the fNIRS acquisition system were transformed to the oxyHb and deoxyHb concentration changes using the modified Beer-Lambert law [29]. A fifth-order Butterworth bandpass filter with 0.02 Hz and 0.4 Hz cutoff frequencies was applied to eliminate the physiological artifacts of low and high frequency such as heartbeat, breathing, and blood pressure changes from the fNIRS signals. The fNIRS signals were smoothed using a moving average filter with a five-point sliding window. The data were segmented into 4 s preceding and 18 s following the movement’s onset mark, the same as the EEG analytic intervals. The interval from 4 to 1 s pre-movement’s onset was chosen for the baseline correction. Then, the data were averaged across the trials and the averaged data were normalized to compare the activations among the channels and the subjects. The entire preprocessing pipeline was implemented through a plug-in analysis software Platform for Optical Topography Analysis Tool [30]. We evaluated the concentration increase in oxyHb because of the fact that the increase in oxyHb is known to be more sensitive to the changes in regional cerebral blood flow and has shown a strong correlation with the BOLD signals [31,32]. The channels over the primary and supplementary motor areas that show significant activation during the motor execution were selected for subsequent data analysis.

### 2.7. EEG and fNIRS Correlation Analysis

The time-course of the hemodynamic response and EEG signals were evaluated by calculating the oxyHb and deoxyHb concentration changes and ERD of the alpha band over the premotor and supplementary motor areas in both hemispheres. The time-course features were demonstrated by plotting the average ERD values of alpha-band and oxyHb concentration changes quantified by their z-scores over subjects. One-tailed Pearson’s correlation analysis between the concentration of oxyHb, deoxyHb, and ERD in the most prominent channels over the bilateral motor cortex areas was evaluated.

### 2.8. Feature Extraction and Classification

Three statistical features were extracted from fNIRS signals including variance, kurtosis, and skewness. On the other hand, time-domain features were extracted from the ERD signal including mean absolute value (MAV), root mean square (RMS), waveform length (WL), and fourth-order autoregressive coefficients. The signals were segmented into a one-second sliding window [33], in the feature extraction stage. Four sets were formed to compare the performance of the single modalities of the fNIRS, EEG, the integrated modality with all channels, and the integrated modality with highly correlated fNIRS and EEG channels. In this study, right and left ankle joint movements were classified. 10-fold cross-validation was applied to 80% of the data to tune the classifier, and then the tuned classifier was tested with the remaining 20% of the data. Support vector machine (SVM) was employed as the classifier to evaluate the performance of the single modality and the integrated modality. The SVM was chosen because of its ability to handle linear boundaries and more complex decision planes that separate the left and right ankle joint movements in this case. A MATLAB function fitcsvm was used with the default settings to train the classifier modal (mdl) and the function predict was used to predict the correct classes. The classifier performance parameters including accuracy, sensitivity, and specificity were evaluated according to the following formulas in Equations (2)–(4):

(2) $$Accuracy=\frac{TP}{TP+TN+FP+FN}$$

(3) $$Sensitivity=\frac{TP}{TP+FN}$$

(4) $$Specificity=\frac{TN}{TN+FP}$$

where

*TP*: true positive rate.

*TN*: true negative rate.

*FP*: false positive rate.

*FN*: false negative rate.

## 3. Results

### 3.1. Hemodynamic Response Analysis Results

**Figure 5:**
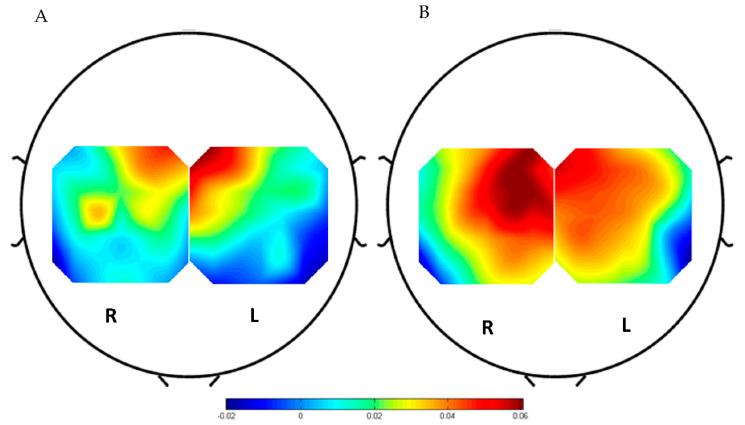
Average topoplot of oxyHb concentration of motor cortex (**A**) during right ankle movement and (**B**) during left ankle movement.

The hemodynamic results showed an increase in oxyHb concentration changes in the left hemisphere during the contralateral right ankle movement. Figure 5A illustrates the topographical map of the oxyHb averaged across all the participants for the right ankle movement. Oxygenation was observed at the left dorsal primary motor cortex (LPMCdr), left somatomotor cortex (LSMC), and left pre-supplementary motor area (pre-SMA). The motor cortex area was covered by channels ch25, ch28, ch32, and ch35. Among all, ch28 and ch32 demonstrated significant activation compared with the nearby channels. On the other hand, Figure 5B demonstrated the topographical map of the hemodynamic response during the left ankle movements. The oxyHb concentration changes showed an increase in the right hemisphere during the contralateral left ankle movement. As expected, the right ventral primary motor cortex (RPMCvr) and right premotor area (RPMA) activated during the motor execution, where the right dorsal primary motor cortex (RPMCdr) is represented by channels ch3, ch7, ch10, and ch14 showed a significant concentration change.

**Figure 6:**
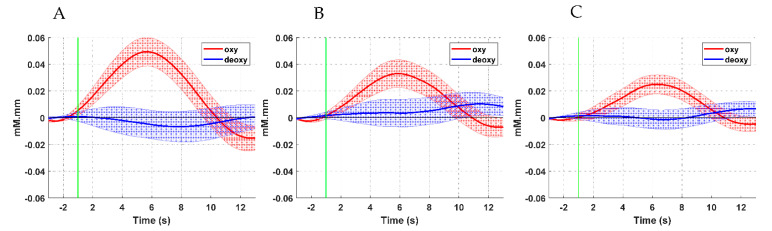
Time course of the averaged oxyHb and deoxyHb of the selected channels with respect to the motor cortex areas during right ankle joint movement: (**A**) LPMCdr, (**B**) LPMCvr, and (**C**) LPMA.

**Figure 7:**
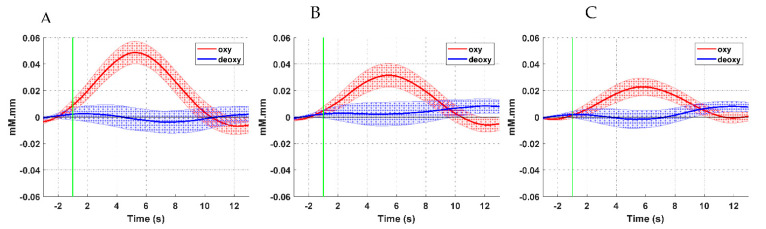
Time course of the averaged oxyHb and deoxyHb of the selected channels with respect to the motor cortex areas during left ankle joint movement: (**A**) RPMCdr, (**B**) RPMCvr, and (**C**) RPMA.

Figure 6 and Figure 7 illustrate the time series of the oxyHb and deoxyHb during both left and right ankle joint movements. In these figures, the oxyHb concentration level decreases immediately after the movement cue for a short period (i.e., the initial dip during 1–2 s) and begins to increase to reach the maximum peak at around 3.8–4.2 s (i.e., the main hemodynamic response) on average. Then, the amplitude declines again as the rest period begins. An initial dip can be defined as an early small reduction in the concentration shift of oxyHB at the neuronal activity locus after stimulus exposure [34,35]. Figure 6 shows that the amplitude of the oxyHb concentration in LPMCdr is higher than those in the left ventral primary motor cortex (LPMCvr) and left pre-motor area (LPMA). Similarly, Figure 7 shows that the amplitude of the oxyHb concentration in the RPMCdr area is higher than the amplitude in RPMCvr and the right primary motor area (RPMA). On the other hand, deoxyHb shows fewer variations in the concentration during the motor execution task for both right and left ankle movements. Therefore, the mean of the LPMCdr and RPMCdr channels was selected for the correlation analysis with ERD of the EEG signals.

### 3.2. ERD Analysis Results

**Figure 8:**
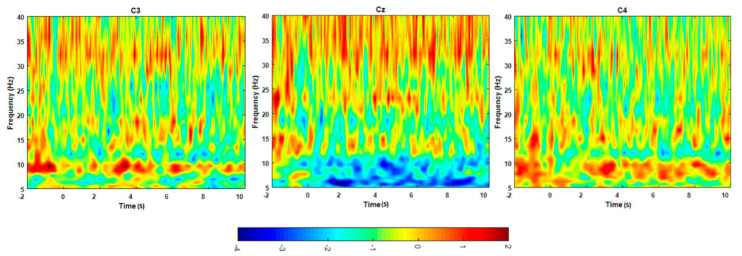
Time frequency map of the average across the participants during the ankle movements; the left map represents left motor cortex area C3, the middle image represents Cz area, whereas the right image represents right motor cortex area C4.

**Figure 9:**
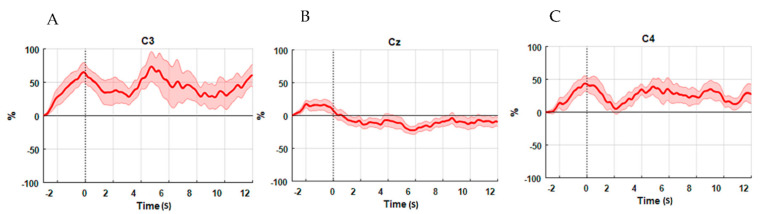
Time course of the ERD/ERS transitions for right ankle joint movements. The EEG signals power of (**A**) C3, (**B**) Cz, and (**C**) C4.

Figure 8 illustrates the average TF plot over the participants of the EEG channels in the right and left motor cortex areas. The blue color indicates the event-related desynchronization, which means a decrease in power, while the red color indicates the increase in power. All changes were evaluated with respect to the baseline period interval between one second before the movement cue signal prior to the movement cue. It can be noted, in Figure 8, that the ERD is pronounced in the frequency range around (8–11) Hz. Thereafter, the ERD signal was smoothed, as shown in Figure 9. The EEG power was evaluated with reference to the period −4 s to −3 s preceding the movement cue and 18 s after the movement cue. The EEG signals exhibit a significant ERD on the Cz electrode (*p* = 0.0064) during the execution ankle joint movement when comparing the power dissipation in the alpha wave before and after movement execution. Meanwhile, the areas of the C3 and C4 electrodes exhibit no ERD, as illustrated in Figure 9. Therefore, EEG electrodes, which neighbor the activated fNIRS channels, reveal a decrease in the average power (desynchronization) of the alpha band. It was then selected for the investigation of the relationship between electrical neural activities and hemodynamic response.

### 3.3. Results of Comparisons between ERD and fNIRS Data

**Figure 10:**
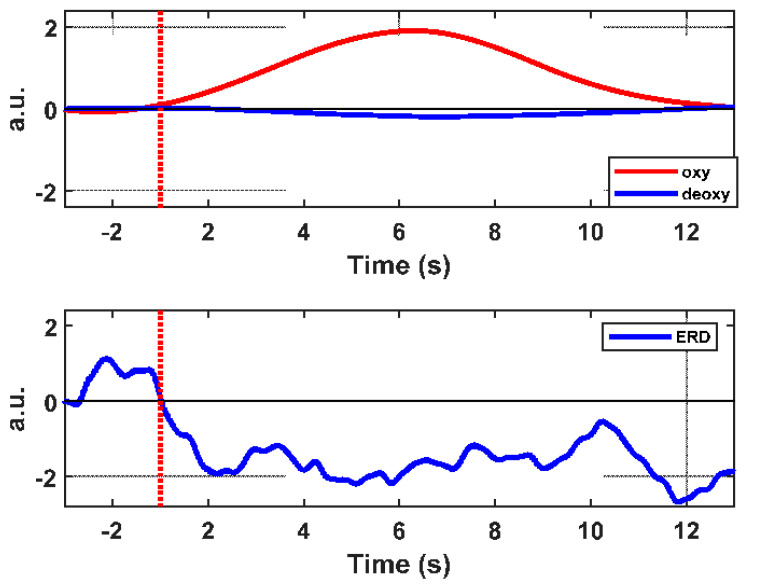
The time evaluation period of the ERD and hemodynamic response, oxyHb in the upper plot red line. ERD represented by the blue line in the lower plot.

As mentioned before, an increase in the oxyHb signal during the motor execution of ankle joint movements is associated with a decline in the power amplitude in the alpha bands. Thus, to quantitively estimate the relationship of the cortical oscillation and hemodynamic response, Pearson’s correlation coefficient was evaluated between the ERD amplitude of alpha oscillations and the oxyHb concentration changes for each subject. Thus, in the central part of the brain, maximal changes of oxyHb and deoxyHb were noticeable, mainly at the optodes that corresponded to the Cz, C1, C2, Cpz, Cp1, Cp2, and FCz electrodes around the LPMCdr and left supplementary motor area (LSMA). These changes were associated with alpha desynchronization oscillations at the relevant EEG electrodes. Figure 10 demonstrates the time-course of the oxyHb and deoxyHb signals in the LPMCdr with the ERD in the Cz electrode. The outcomes revealed that the increase in oxyHb in channels (ch25, ch28, ch32, and ch35) in which the signal change was most prominent in the LPMCdr area was significantly correlated with the change in the ERD on Cz and Cpz one-tailed Pearson’s correlation, r = −0.64061, *p* < 0.001 and r = −0.63779, *p* < 0.001, respectively. No such correlation was found in the right hemisphere where the correlation between RPMCdr with C4 is r = −0.08489 and *p* = 0.052380. Table 1 shows the correlation coefficients with the *p*-value for the EEG electrodes with the averaged fNIRS channels (ch25, ch28, ch32, and ch35) in the LPMCDr area. Similarly, during the left ankle joint movements, a significant correlation between fNIRS averaged channels (ch3, ch7, ch10, and ch14) in the RPMCdr area with neighboring EEG electrodes were found (refer to Table 2). On the other hand, Table 3 and Table 4 demonstrated the positive correlation coefficients between the deoxyHb and alpha band in the RPMCdr area. Where the highest correlations were found in the Cz, Cpz, and Cp1, respectively, during the right ankle movement. Meanwhile, Cp2 showed the highest correlation of r = 0.49299, *p* = 2.31 × 10^−7^ during the left ankle movements. Additionally, the delay time between the hemodynamic response (oxyHb) and electrophysiological signals varied on average by 4.18 ± 2.418 s in the LPMCdr and foot area (Cz).

### 3.4. Results of Movement Classification

**Figure 11:**
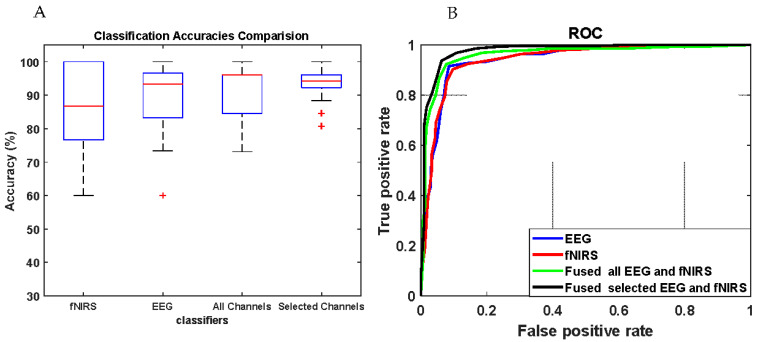
(**A**) Classification accuracies of ankle joint movements based on the single and bi-modilties; the (+) symbols represent the outliers’ values. (**B**) ROC for EEG, fNIRS, and integrated EEG and fNIRS.

**Figure 12:**
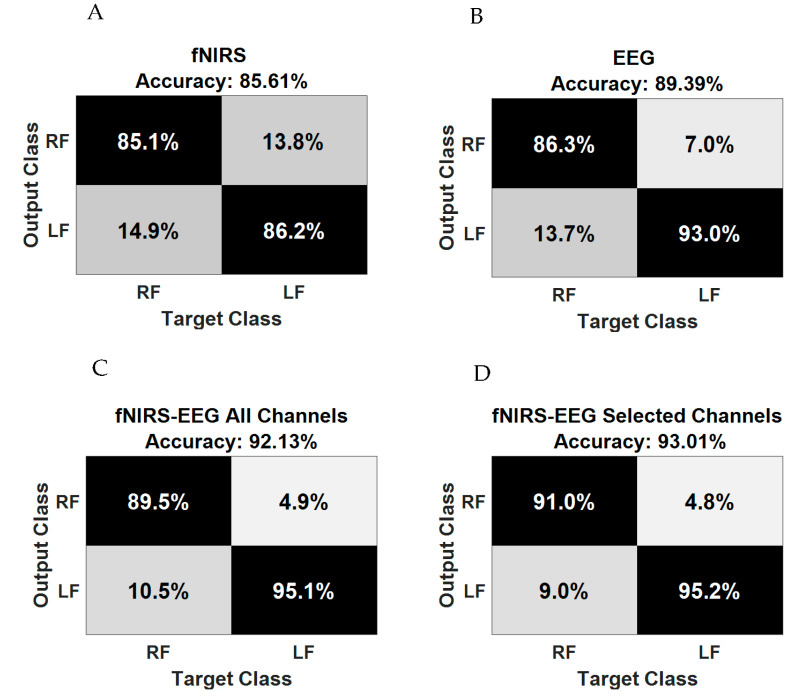
Confusion matrix of (**A**) fNIRS alone, (**B**) EEG alone, (**C**) fNIRS–EEG based on all channels, and (**D**) fNIRS–EEG based on selected channels.

Figure 11 and Table 5 illustrate the overall classification performance of the right and left ankle joint movements using the three modalities: fNIRS, EEG, and integrated EEG and fNIRS. The average classification accuracies of the two ankle joint movements are illustrated in the confusion matrices in Figure 12. The correct and incorrect classifications are shown by the main diagonal and off-diagonal records, respectively. Particularly, the average classification accuracy of the fNIRS modality alone is 85.61 ± 13.42%, whereas the average classification accuracy of the EEG modality alone is 89.39 ± 10.67%. On the other hand, the classification accuracy of the integrated modality fNIRS–EEG (or fused model) based on all recorded channels is 92.13 ± 8.09%, whereas the classification accuracy of the integrated modality fNIRS–EEG based on the correlated channels is 93.01 ± 5.60%.

The ROC curves are shown in Figure 11B. The integrated modality with selected fNIRS and EEG channels shows a higher level of performance in comparison with single modalities (EEG/fNIRS alone). Furthermore, it slightly outperforms the similar model fused with all EEG and fNIRS channels. The results reveal that the integrated modality based on the correlated channels performed better, with classification accuracy increased by ~7% with respect to the fNIRS and ~3% with respect to the EEG modality alone. Table 4 lists the classification performance results including average classification accuracy, sensitivity, and specificity. The enhancement of using integrated modality fNIRS and EEG based on the correlated channels is statistically significant (*p* = 0.0018 and *p* = 0.0083) with individual fNIRS and EEG modality, respectively. There are improvements not only in the classification accuracy but also in terms of channel reduction.

## 4. Discussion

The current study aims to investigate and exploit the relationship between the electrical neural activity and hemodynamic response based on EEG and fNIRS simultaneous recordings to better detect ankle joint movements. According to the prior hypothesis, an increase of the oxyHb in the motor cortex area is associated with the power spectrum of the brain rhythm in the alpha band (8–11) Hz. The outcomes of this study reveal that the dependency between ERD in the alpha and oxyHb is negatively correlated and the highest correlation coefficients were found in the Cz and Cpz electrodes. The negative correlation behavior of this study is consistent with the previous research. Lachert et al. [23] investigated the relationship between EEG rhythms in alpha and beta bands and hemodynamic response during a motor task (self-paced right finger movements), and they found that the highest correlation coefficient between alpha rhythm (8–13) Hz and oxyHb in the hand area C3 is (r^2^ = −0.69, *p* < 0.0001) and Hb and beta (r^2^ = −0.54) rhythms [23]. During the self-paced button press task, Zama and Shimada [17] demonstrated the correlation between the readiness potentials and oxyHb. The finding revealed that the readiness potential (RP), which is a negative slow potential slop arising during motor preparation on the hand area C3 in the extended 10–20 system appeared around 1000 ms preceding the movement onset. During the motor preparation, an increase in oxyHb concentration changes in the premotor cortex is also confirmed by fNIRS, which results in a significant correlation between the amplitude of the RP and the change in oxyHb concentration (Pearson’s correlation r^2^ = 0.235, *p* = 0.03).

Alpha oscillations occurring in the central portion of the brain are the representation of neural populations’ synchronous activities. The contribution of synchronous neurons to the alpha power amplitude is proportional to their numbers N, while it is proportional to √N in non-synchronous populations [36]. In the present study, ERD in the alpha oscillation-band (8–11) Hz occurs in the PMCdr and supplementary motor area (SMA); however, a significant ERD occurs in the Cz area during the right ankle movements. ERDs (decreasing in the average power) were observed in the Cz and Cpz electrodes close to the foot representation area in the alpha band during the ankle joint movements from all subjects, whereas only weak ERS could be noticed over the ipsilateral side in the C4 and C3 areas, as illustrated in Figure 9. These facts are derived from a decrease in the synchrony of the underlying neuronal populations during motor execution (ERD), as reported in [37].

The fNIRS analysis shows an increase of the oxyHb concentration changes in PMCdr and SMA. Although the contralateral activations were more pronounced during the right ankle joint movements, ipsilateral activation was also observed, as illustrated in Figure 4. On the other hand, bilateral activations were observed during the left ankle joint movement where contralateral activations were more pronounced. However, the correlation between the RPMCdr and C4 is r = −0.08489 (*p* = 0.052380). Contralateral and ipsilateral activation depends on the dominant and nondominant limb movements. Previous studies [38,39,40] in brain activities’ lateralization during upper and lower limb joints movement showed that bilateral activation is more pronounced during nondominant than during dominant unilateral movement, especially in right-handed subjects [38,41,42]. In line with the previous findings, this work showed that bilateral activations were more pronounced during the non-dominant ankle joint movement than the movement of the dominant ankle, as demonstrated in Figure 5B. The observed distribution of oxyHb concentration changes during the ankle movements does not reflect only the involved motor cortical systems, but also a rather unspecific activation of somatosensory cortical areas. Moreover, Obrig et al. [39] suggested that bilateral effects might be due to the characteristics of the human brain metabolism, in addition to the oxyHb being strongly influenced by systemic changes in cerebral blood flow [40].

The current study determined a correlation between the EEG and fNIRS signals in the motor cortex during motor execution of ankle joint based on the hemodynamic response. Our finding of the specific correlation of ERD in the alpha band (8–11) Hz with a hemodynamic response in the motor cortex area indicates that the fNIRS signal represents the neural electric excitation process [43,44,45,46]. However, this finding should be properly considered as the correlation does not mean necessarily that the same neural activity was captured by the EEG and fNIRS. In addition, the experimental results of this study reveal that integrating fNIRS with EEG signals would improve the classification accuracy of the ankle joint movements. When compared with standalone EEG and fNIRS, combined fNIRS–EEG systems showed improved sensitivity and specificity with all or selected fNIRS–EEG channels. The sensitivity was improved by ~8% and ~5% when comparing the integrated modality with fNIRS and EEG alone, respectively. Moreover, the specificity was improved by ~9% and ~2% when comparing the integrated modality with fNIRS alone and EEG alone, respectively. The proposed channel selection approach helps to take advantage of the strength of each modality for the movement’s recognition analysis. Moreover, these investigations contribute to a better understanding of neurovascular integration and bringing promise to the brain-computer interface design. Additionally, these outcomes can help in finding the most related cortical areas involved in the movement task. In particular, the correct signals for the BCI cannot be attained if the inappropriate cortical areas are selected. If we know the exact location of the brain to be monitored, the burden of placing heavy caps of electrodes for activity detection can be avoided. The problem in this context is how to select a small brain region so that a limited number of electrodes can be used to control the BCI system. Furthermore, these promising outcomes reveal the potentials of the integration-based approach for accelerating the pace of future development of a BCI for the rehabilitation of the lower limbs. Additionally, the developed EEG and fNIRS systems could contribute to the proliferation of bio-robotics assistive devices that can support the movements and result in enhancing the quality of human life.

Some limitations associated with the fNIRS signals include the bilateral effects in the hemodynamic response. Besides the effects of dominant and nondominant limbs, systemic hemodynamic changes also affect the hemodynamic response [47]. Systemic hemodynamic changes are caused by many potential factors, such as changes in heart rate, blood pressure, and respiration. During motor functions, including finger tapping, heart rates increase [48]. These effects can be minimized by implementing some post signal processing such as general linear model (GLM) with systemic regression techniques and depth-resolved techniques as reported in [48,49]. Moreover, inherited hemodynamic delay limits the utilizing of fNIRS in BCI applications. Furthermore, when integrating the two modalities, the key drawback arises from the shared scalp surface and the presence of wires/fibers, particularly if a large number of sensors is used. A good fNIRS signal relies on mechanical rigidity and proper optode coupling with the scalp surface, whereas EEG normally requires the use of gels to reduce impedance. Moreover, this study possesses some limitations that need to be addressed. The experiments in this work were carried out with healthy subjects. Therefore, this work should be extended to include disabled subjects. Additionally, the current study investigates the actual movements; however, the imagination scheme is also crucial in the development of the BCI solutions.

## 5. Conclusions

Multimodal neuroimaging methods today have a significant influence on neuroscience research. This study investigated and exploited the neurovascular coupling correlation between EEG and fNIRS simultaneous measurements for lower limb movement detection. The oxyHb was extracted as an indicator of the concentration changes of the hemodynamic response, and the ERD signal was evaluated in the alpha band (8–11) Hz. Then, Pearson’s correlation coefficient was implemented to quantitively estimate the dependency between oxyHb and ERD signal during the ankle joint movements. The results demonstrated that the hemodynamic response is negatively correlated with the ERD signal in the alpha brain oscillation band. The highest correlation coefficient was observed between fNIRS (ch28, ch25, ch32, ch35) with Cz and Cpz EEG channels for the right ankle joint movements. The correlated channels were then used for ankle joint movement classification in both modalities. The statistical analysis findings show that the integrated modality based on the correlated channels offers significantly higher accuracy of the ankle joint classification of 93.01 ± 5.60% (*p* < 0.01) compared with the single modality.

## Figures and Tables

**Table 1:** Correlation coefficients of the alpha wave with oxyHb in the LPMCdr cortical area during right flexion (RF).

	EEG Electrodes					
	Cz	C1	Cpz	Cp1	FCz	FC1
rho	−0.64061	−0.56344	−0.63779	−0.63173	−0.46765	−0.37868
*p* value	7.54 × 10^−4^	1.59 × 10^−2^	1.04 × 10^−4^	5.49 × 10^−10^	1.09 × 10^−4^	4.47 × 10^−4^

**Table 2:** Correlation coefficients of the alpha wave with oxyHb in the RPMCdr cortical area during left flexion (LF).

	EEG Electrodes					
	Cz	C2	Cpz	Cp2	FCz	FC2
rho	−0.45656	−0.46217	−0.42304	−0.40145	−0.44859	−0.37647
*p* value	9.97 × 10^−5^	5.95 × 10^−3^	6.96 × 10^−3^	1.54 × 10^−2^	2.62 × 10^−2^	1.26 × 10^−2^

**Table 3:** Correlation coefficients of the alpha with deoxyHb in the LPMCdr cortical area during RF.

	EEG Electrodes					
	Cz	C1	Cpz	Cp1	FCz	FC1
rho	0.52406	0.43188	0.51047	0.50175	0.35362	0.35397
*p* value	1.18 × 10^−6^	5.38 × 10^−5^	2.11 × 10^−5^	3.31 × 10^−7^	2.08 × 10^−4^	8.66 × 10^−7^

**Table 4:** Correlation coefficients of the alpha wave with deoxyHb in the RPMCdr cortical area during LF.

	EEG Electrodes					
	Cz	C2	Cpz	Cp2	FCz	FC2
rho	0.42972	0.38649	0.43163	0.49299	0.35336	0.19462
*p* value	5.69 × 10^−5^	3.94 × 10^−4^	3.49 × 10^−6^	2.31 × 10^−7^	7.59 × 10^−3^	1.47 × 10^−3^

**Table 5:** Classification performance.

	Modalities			
	fNIRS	EEG	fNIRS and EEG All Channels	fNIRS and EEG Selected Channels
Accuracy %	85.61	89.39	92.13	93.01
Sensitivity %	86.04	92.49	94.49	94.98
Specificity %	85.26	87.16	90.05	91.36

## Data Availability

The data sets are not publicly available.
